# Unplanned transfer to acute care during inpatient geriatric rehabilitation: incidence, risk factors, and associated short-term outcomes

**DOI:** 10.1186/s12877-024-05081-3

**Published:** 2024-05-24

**Authors:** Sofia Fernandes, Christophe Bula, Hélène Krief, Pierre-Nicolas Carron, Laurence Seematter-Bagnoud

**Affiliations:** 1 Service of Geriatric Medicine and Geriatric Rehabilitation, Lausanne University Hospital and University of Lausanne (CHUV), Lausanne, 1011 Switzerland; 2 Centre for Primary Care and Public Health (Unisanté), Department of Epidemiology and Health Systems, University of Lausanne, Lausanne, Switzerland; 3 Emergency Department, Lausanne University Hospital and University of Lausanne (CHUV), Lausanne, 1011 Switzerland

**Keywords:** Rehabilitation, Geriatrics, Acute transfers, Risk factors, Infections

## Abstract

**Background:**

Information is scarce on unplanned transfers from geriatric rehabilitation back to acute care despite their potential impact on patients’ functional recovery. This study aimed 1) to determine the incidence rate and causes of unplanned transfers; 2) to compare the characteristics and outcomes of patients with and without unplanned transfer.

**Methods:**

Consecutive stays (*n* = 2375) in a tertiary geriatric rehabilitation unit were included. Unplanned transfers to acute care and their causes were analyzed from discharge summaries. Data on patients’ socio-demographics, health, functional, and mental status; length of stay; discharge destination; and death, were extracted from the hospital database. Bi- and multi-variable analyses investigated the association between patients’ characteristics and unplanned transfers.

**Results:**

One in six (16.7%) rehabilitation stays was interrupted by a transfer, most often secondary to infections (19.3%), cardiac (16.8%), abdominal (12.7%), trauma (12.2%), and neurological problems (9.4%). Older patients (AdjOR_age≥85_: 0.70; 95%CI: 0. 53–0.94, *P* = .016), and those admitted for gait disorders (AdjOR: 0.73; 95%CI: 0.53–0.99, *P* = .046) had lower odds of transfer to acute care. In contrast, men (AdjOR: 1.71; 95%CI: 1.29–2.26, *P* < .001), patients with more severe disease (AdjOR_CIRS_: 1.05; 95%CI: 1.02–1.07, *P* < .001), functional impairment before (AdjOR: 1.69; 95%CI: 1.05–2.70, *P* = .029) and at rehabilitation admission (AdjOR: 2.07; 95%CI: 1.56- 2.76, *P* < .001) had higher odds of transfer. Transferred patients were significantly more likely to die than those without transfer (AdjOR 13.78; 95%CI: 6.46–29.42, *P* < .001) during their stay, but those surviving had similar functional performance and rate of home discharge at the end of the stay.

**Conclusion:**

A significant minority of patients experienced an unplanned transfer that potentially interfered with their rehabilitation and was associated with poorer outcomes. Men, patients with more severe disease and functional impairment appear at increased risk. Further studies should investigate whether interventions targeting these patients may prevent unplanned transfers and modify associated adverse outcomes.

## Introduction

Older patients admitted to inpatient rehabilitation face a significant risk of acute complications during their stay, some requiring their transfer back to acute care [1–4]. Studies on the incidence, causes, and consequences of these transfers remain however limited [5]. Notably, several studies did not focus on unplanned transfers only [6, 7], while others included patients admitted to rehabilitation for selected conditions [8–17], or younger than 65 years [18–20], or were performed in skilled nursing facilities and not inpatient rehabilitation setting [21–23]. Four studies specifically investigated unplanned transfers from geriatric rehabilitation back to acute care [1–4], with widely variable rates ranging from 4 to 22%. Significant variations are also observed in the causes of unplanned transfers reported in these studies. For instance, infectious or cardiac complications accounted for 14% to 44% and 8% to 25% of the transfers, respectively [1–4], seriously hindering decisions about which specific preventative interventions to propose [24].

Similarly, information about patients’ characteristics associated with an increased risk of transfer is heterogeneous [24]. For instance, the only study of our literature search that investigated the potential role of comorbidity did not find any significant association with unplanned transfers [4]. Moreover, findings were inconsistent for some markers of poor health, notably polypharmacy, and impaired functional status, and no association was reported for age and sex [2, 4]. Clarifying these discordant findings and identifying additional patients’ characteristics carrying a risk for rehospitalization due to some conditions may allow to improving and better targeting strategies to prevent such complications, and manage at best potential transfers to acute care.

Finally, although recognition of acute problems ending in transfers allow their timely management, an additional concern about unplanned transfers from rehabilitation back to acute care stems from the potential negative influence on patients’ functional recovery, beyond the acute health problem that triggered the transfer. For example, a study highlighted that transferred older patients had poorer functional recovery and were less likely to be discharged to their home than those without transfer [2]. Outcomes such as functional recovery and discharge location remain however largely understudied in patients transferred to acute care from inpatient rehabilitation care. Providing additional information on the magnitude of these issues appears therefore important.

The present study aimed to address these gaps in determining the incidence rate and main causes of unplanned transfers from hospital-based geriatric rehabilitation to acute care. A second aim was to compare the characteristics and short-term outcomes in patients with and without unplanned transfer. Specifically, the functional performance, discharge destination, and death rate at the end of the rehabilitation stay were compared in patients with and without unplanned transfer.

## Methods

### Setting and population

This retrospective study included patients aged 65 years or more consecutively admitted between June 1, 2018 and June 30, 2020 to the 95-bed geriatric rehabilitation unit of an academic hospital in Switzerland. In this country, patients are essentially transferred from acute care to inpatient rehabilitation when unable to return directly to their home because of functional and mobility impairments. Indeed, most patients (> 95%) enrolled in the present study were admitted after a stay in acute care for surgery (e.g., hip fracture, abdominal surgery), falls without injury, acute heart failure, respiratory, or digestive conditions, or neurological diseases (e.g., Parkinson’s disease, stroke, …), while only few were admitted directly from home or nursing home. After an initial comprehensive geriatric assessment, the usual rehabilitation program is conducted by a multidisciplinary team that provides 30-min daily physical therapy sessions, biweekly occupational therapy sessions, and daily nursing rehabilitation training in activities of daily living (ADLs), together with individualized interventions (e.g., nutritionist, clinical pharmacist, psychologist, etc.) targeted according to patient’s needs and goals of care. The unit has a permanent medical coverage, and the ability to deliver intravenous treatments and oxygen supplementation, among others, but does not have on-site radiology service.

Usually, a transfer is considered in case of clinical instability, need for intensive therapy (e.g., continuous i.v. treatment) or monitoring (e.g., cardiac monitoring), and failure in at least two organs/systems (e.g., cardiac and respiratory).

### Unplanned transfer

An unplanned transfer was defined as an episode of unexpected clinical deterioration that required referral to acute care (usually through the emergency department, ED), independent of whether or not a subsequent admission to an acute care ward occurred. Planned transfers to perform elective investigations or interventions were excluded. Unplanned transfers were further qualified as “early” or “later” if occurring within or after the first 72 h after admission to rehabilitation, respectively, a cut-off used in other studies [13, 18, 19].

Information on each transfer to acute care, including the exact dates of transfer and, when applicable, of readmission to rehabilitation, was retrieved from the hospital administrative database.

To determine the incidence rate of unplanned transfers to acute care, a single reviewer (S.F.) performed a content analysis of the discharge summary from each rehabilitation stay that ended by a transfer to acute care. For each confirmed unplanned transfer, a cause was determined after reviewing the discharge summaries from: a) the rehabilitation stay (at the time of the transfer and/or at rehabilitation discharge in patients who returned to rehabilitation after their transfer); b) the ED, or the acute unit for patients admitted after their transfer. The cause of transfer was defined as the diagnosis eventually identified after clinical investigations in acute care, if different from the initial diagnosis that motivated the transfer. To ensure quality of data abstraction, a senior physician (L.S.-B.) independently assessed the nature (planned vs unplanned) and the cause of the transfer for the first fifty cases, blinded to first reviewer’s results. Thereafter, the senior physician performed a systematic review in all cases considered difficult by the first reviewer and quality checks in about one in ten randomly chosen cases.

Overall, causes of transfer were grouped into eight categories: infections, trauma, cardiac, gastro-intestinal, respiratory, orthopedic, neurological, and miscellaneous problems.

### Short-term outcomes

Information on in-hospital death and discharge destination (home vs nursing home and other places) at the end of the rehabilitation stay (for survivors and patients who did came back to rehabilitation after their transfer) were retrieved from the hospital administrative database. Functional status at discharge was assessed by the Functional Independence Measure (FIM) total score (i.e. total of 126) [25].

### Baseline covariates

Information was collected at rehabilitation admission on age, sex, living situation (alone vs not), premorbid performance in Katz basic ADL (BADL [26]), as well as in and Lawton’s instrumental ADL(IADL [27]), in-home care need, location prior to rehabilitation admission (i.e., acute hospital vs home or nursing home), depressive symptoms (Mini-Geriatric Depression Scale [28]), cognitive impairment (defined as a Mini-Mental State Exam score < 24/30 [29] or Montreal Cognitive Assessment score < 26/30 [30] or Mini-COG score < 3/5 [31]), BADL impairment at admission (defined as a score of 30/42 on FIM self-care items (eating, grooming, bathing, getting upper/lower body dressed, toileting) [25], comorbidity using the Cumulative Illness Rating Scale [32, 33] (CIRS), and main admitting diagnosis to rehabilitation.

### Statistical analysis

#### Analysis of stays

Transfer rate and their causes were computed for all identified stays and according to early (i.e., within the first 72 h) vs later transfers as a subgroup analysis. An analysis was performed to assess the proportion of unplanned transfers which causes were lied within the same diagnosis category that motivated the initial admission to rehabilitation. Finally, the length of stay in acute care following the transfer was computed.

#### Analysis of patients’ characteristics and short-term outcomes

Comparisons of characteristics and short-term outcomes in patients with and without transfer were performed at the patient’s (and not stay) level. For patients admitted twice or more in rehabilitation during the study period, only the first stay was considered. Characteristics of patients with and without unplanned transfer were compared in bi-variable. Then, a multivariable logistic regression model examined the association between patients’ characteristics and having been transferred, adjusting for variables significantly associated with being transferred in bivariable analyses.

Short-term outcomes (in-hospital death, discharge to their own home, and functional status at discharge) were first compared in patients with and without unplanned transfer in bivariable analysis. Information on discharge destination and functional status at discharge was not available for patients who died during hospitalization (*n* = 63), and for those who did not come back to rehabilitation after an unplanned transfer (*N* = 82), leaving a sample of *N* = 1,784 (92.5% of total sample) for these analyses.

Multivariable logistic regression analyses of the association between transfer for acute care and short-term outcomes (functional status at discharge, death and discharge location (home vs other)) were then performed. Adjustment variables were identified from literature and previous knowledge, they included socio-demographics (age, sex, living situation), mental health (cognitive and affective impairment), comorbidity (CIRS), and functional status (FIM) at rehabilitation admission.

The absence of multicollinearity was verified in all models.

Analyses were performed using Stata 16 (StataCorp, Texas).

### Availability of data

Data used in this study were retrieved from the hospital administrative database as regards information about transfers to acute care and patients’ characteristics, while reasons of transfers were determined from discharge summaries. All data were merged into a single database for analytical purposes. Data are not publicly available, but might be available from the corresponding author on reasonable request.

### Ethical considerations

The study was approved by the local Human Research Ethical Committee (Commission cantonale (VD) d’éthique sur la recherche sur l’être humain (CER-VD), N°2020–01866). According to the Swiss law on research on human beings (LRH), article N°34, the Human Research Ethical Committee allowed the reutilization of routinely collected data for patients who provided written informed consent and for patients were duly informed about the potential use of their data for research projects but did not fill the consent form. Patients who refused the retrospective use of their routine data for research projects (*N* = 375) were excluded.

## Results

**Fig. 1:**
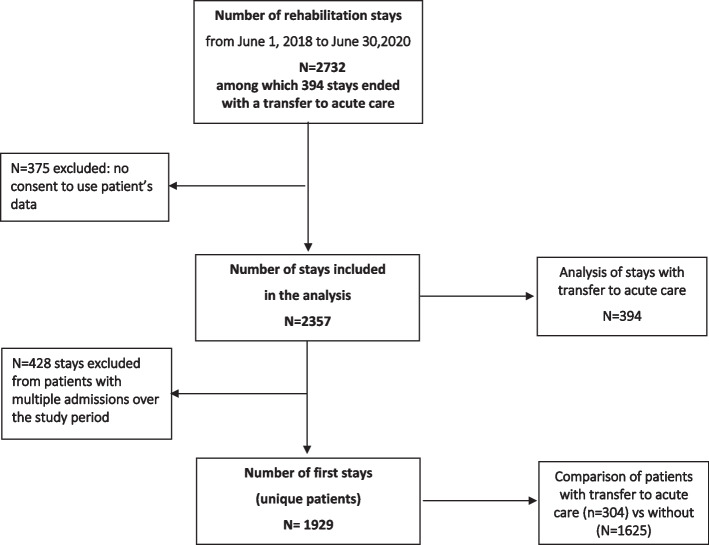
Flow diagram of the selection of stays and unique patients included in the analyses

Overall, 2,732 rehabilitation stays occurred over the study period (Fig. 1), from which 375 were excluded because of patients’ refusal to use their data for research, leaving 2,357 stays for the analysis. Analyses at patient’s level were conducted on a sample of 1,929 unique patients after exclusion of 428 stays resulting from multiple admissions (18.2%).

### Incidence rate, timing, and causes of transfers

**Fig. 2:**
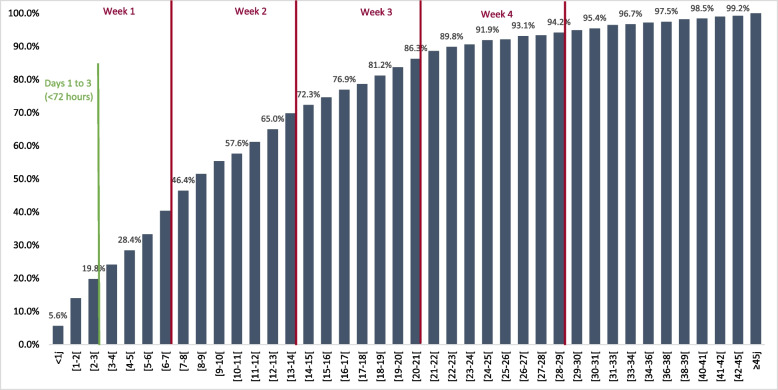
Cumulative incidence of unplanned transfers, in days from admission to rehabilitation

Among 2,357 stays included in this analysis, 394 (16.7%) were interrupted by at least one unplanned transfer. Of these, 351 (14.9% of the total sample) were stays with a single transfer, and 43 (1.8% of the total sample) were stays with two (*N* = 36) or more (*N* = 7) transfers (maximum four transfers). Figure 2 depicts the cumulative incidence of unplanned transfers over time and shows that one in five (19.8%) qualified as “early” transfers (i.e. within 72 h after admission to rehabilitation). About half (46.4%) of the transfers occurred within the first week of rehabilitation, and almost three quarters (72.3%) within the first two-week period.

Table 1 shows the distribution of the main causes of unplanned transfers by diagnostic category. Infections were the most frequent causes for transfer (19.3%), with pneumonia (including 13 cases of Covid-19), sepsis, and pyelonephritis as top three specific diagnoses. Cardiac (16.8%), gastro-intestinal (12.7%), trauma (12.2%, caused mostly by falls), and neurological problems accounted for about one in six to one in ten transfers.

**Table 1:** Main causes of unplanned transfers to acute care (*N* = 304)

Diagnostic category	Specific diagnoses (first three)	Percent
Infections	Pneumonia (including COVID-19, *n* = 13), sepsis, pyelonephritis	19.3%
Cardiac problems	Heart failure, acute coronary syndrome, arrhythmia	16.8%
Gastro-intestinal problems	Gastrointestinal bleeding, ileus, cholecystitis	12.7%
Trauma	Fall, other trauma	12.2%
Neurological problems	Delirium, acute stroke, transient ischemic attack	9.4%
Orthopedic problems	Post-orthopedics complications (except infections)	7.9%
Respiratory problems	COPD, pulmonary embolism, pleural effusion	6.9%
Miscellaneous^a^		14.8%
Total		100.0%

^a^Includes (from most to less frequent): angiologic, rheumatologic, uro-nephrologic, metabolic, oncologic, dermatologic, otorhinolaryngologic, drugs, psychiatric, surgical and uncertain conditions/problems

The distribution of these diagnostic categories slightly differed between “early” vs “later” transfers, although not significantly (*P* = 0.150). While the most frequent causes of “early” transfers originated from cardiac (24.4%) and gastro-intestinal (16.7%) problems, followed by infections and trauma (12.8% each), “later” transfers were mostly due to infections (21.0%), followed by cardiac (14.9%), trauma (12.0%) and gastro-intestinal (11.8%) problems.

Overall, 42.9% of unplanned transfers were due to a cause belonging to the same diagnostic category than the diagnosis that motivated the initial admission to rehabilitation. However, this proportion varied by initial diagnostic category. It was highest for orthopedic (87.1%, mostly prosthetic infection or dislocation in patients admitted for rehabilitation post-arthroplasty), trauma (54.2%, most frequently after a fall), and respiratory (48.1%, mostly relapsing bronchopneumonia) problems. Contrariwise, only about a third of transfers due to abdominal (32.0%) and cardiac (34.8%) complications occurred in patients admitted to rehabilitation with such a problem.

In more than two-thirds of unplanned transfers (273/394, 69.3%), the patient eventually returned to rehabilitation, within 24 h in half of these cases (136/273, 49.8%), and within 3 days in three-quarters of these cases (203/273, 74.3%).

### Comparison of characteristics in patients with and without transfers

Among the 1,929 unique patients included in this analysis (Fig. 1), 304 (15.8%) experienced one transfer to acute care during their first stay (Table 2). These patients were younger, more often men, and were more functionally impaired before (instrumental ADLs), as well as at the time (basic ADLs) of admission to rehabilitation, but lived less frequently alone than those without transfer. Transferred patients also had higher comorbidity (CIRS) than patients without transfer, and more frequently suffered from heart and respiratory problems. As regards their admitting diagnoses, gait disorders, fracture, and osteoarthritis were less frequent among patients with acute transfer than without transfer (all *P*-values < 0.05).

**Table 2:** Comparison of patients with and without unplanned transfer to acute care

	All patients	Any transfer during the first stay ?		*P*-value*
		Yes	No	
	*N* = 1929	*N* = 304	*N* = 1625	
	(100%)	(15.8%)	(84.2%)	
**Baseline characteristics (before hospital admission)**				
Age, mean [± SD]	83.8 [± 7.6]	82.7 [± 7.9]	84.0 [± 7.5]	.005
Men (%)	36.0	49.7	33.5	< .001
Living alone (%)	62.8	54.0	64.4	.001
BADL impairment^a^ (%)	47.4	47.3	47.4	.980
IADL impairment^b^ (%)	82.2	87.7	81.2	.007
In home care recipient (%)	63.4	61.0	63.9	.341
Acute care stay in the same hospital (%)	85.6	87.8	85.2	.226
**Characteristics at rehabilitation admission**				
Depressive symptoms^c^ (%)	35.6	38.9	35.0	.216
Cognitive impairment^d^ (%)	42.3	44.8	41.9	.382
BADL impairment from FIM^e^ (%)	42.0	58.0	39.4	< .001
CIRS ^f^mean [± SD]	18.9 [± 5.7]	20.8 [± 5.7]	18.6 [± 5.6]	< .001
Admitting diagnosis (%)				
Gait disorders	30.4	25.0	31.5	.035
Fracture	17.4	16.8	17.5	
Post-orthopedic surgery	5.8	3.6	6.2	
Heart failure	4.4	5.3	4.2	
Pneumonia, COPD	3.8	4.9	3.6	
Miscellaneous	38.2	44.4	37.1	

^*^P-value from Pearson chi-squared test (categorical variables) or Student’s t-test (continuous variables)

^a^Katz Basic Activities of Daily Living (ADL) score < 6/6

^b^Lawton Instrumental ADL score < 8/8

^c^Mini-Geriatric Depression Scale score > 0/4

^d^Mini-Mental State Examination score < 24/30 or Montreal Cognitive Assessment score < 26/30 or Mini-COG score < 3/5

^e^FIM score < 30/42 on self-care items (eating, grooming, bathing, getting upper/lower body dressed, toileting)

^f^*CIRS* Cumulative Ilness Rating Scale; scores range from 0 to 56, with higher scores indicating higher comorbidity

**Fig. 3:**
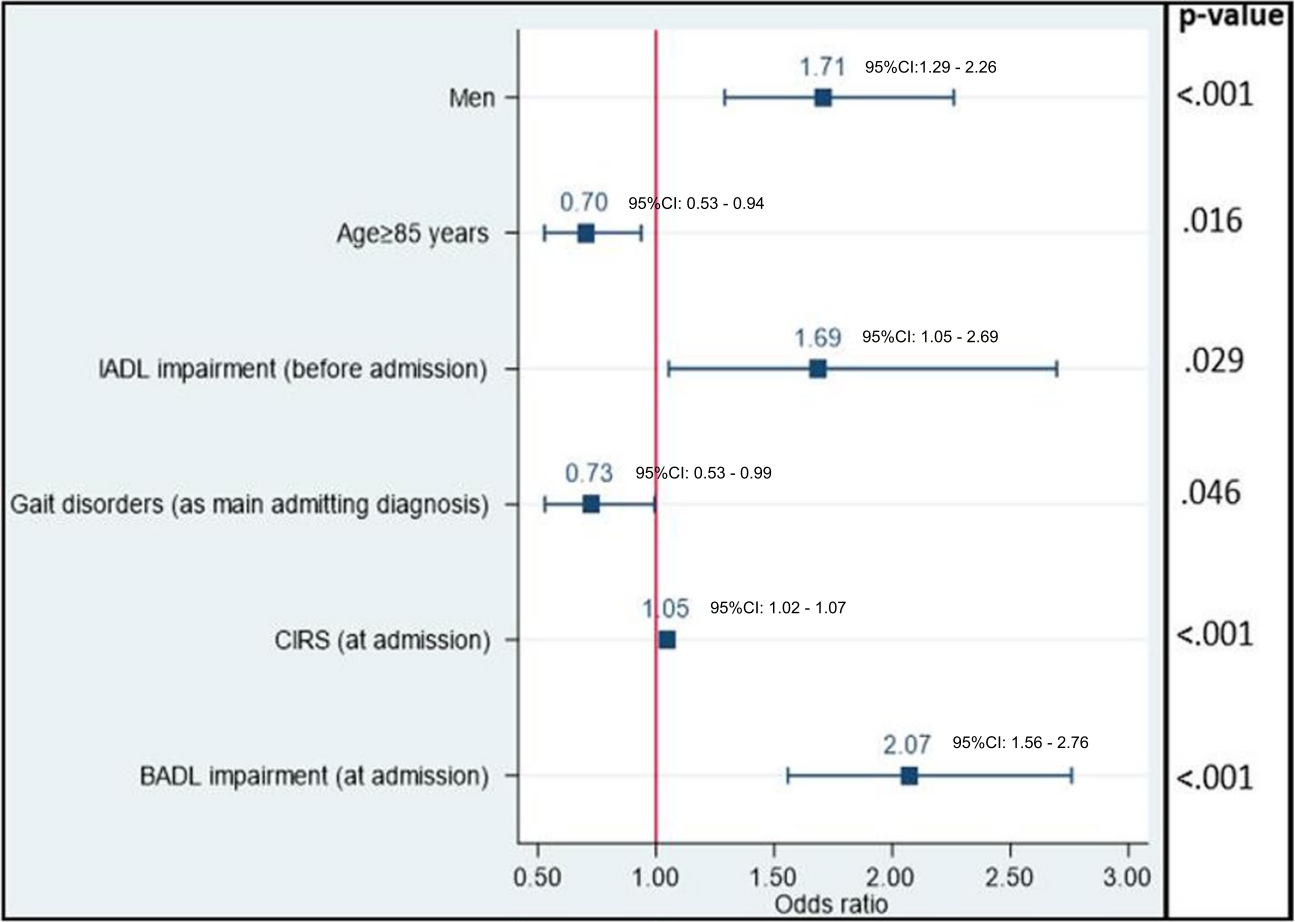
Results of multivariable analysis examining the association between patients’ characteristics and unplanned transfer to acute care

In multivariable analysis (Fig. 3), older patients (adjOR_age≥85_: 0.70; 95%CI: 0. 53–0.94, *P* = 0.016), and those admitted for gait disorders (AdjOR: 0.73; 95%CI: 0.53–0.99, *P* = 0.046) had lower odds of transfer. Inversely, men (AdjOR: 1.71; 95%CI: 1.29–2.26, *P* < 0.001), patients with higher comorbidity (AdjOR_CIRS_: 1.05; 95%CI: 1.02–1.07, *P* < 0.001), IADL impairment prior to hospitalisation (AdjOR: 1.69; 95%CI: 1.05–2.70, *P* = 0.029), and BADL impairment at rehabilitation admission (AdjOR: 2.07; 95%CI: 1.56- 2.76, *P* < 0.001) remained at higher odds of transfer.

### Short-term outcomes

Patients with unplanned transfers were more likely to die (12.5% vs 1.5%, *p* < 0.001), an association that remained significant after adjustment for socio-demographic and health variables (adjOR: 13.8; 95%CI: 6.45- 29.41, *P* < 0.001). The analysis of discharge destination (excluding patients who died and those who did not return to rehab after their transfer) did not show a difference in the proportion of patients discharged to their home between those with and without transfer (81.0% vs 85.6%, *P* = 0.098). Finally, patients with transfer had poorer functional status at discharge than patients without transfer (mean FIM score at rehabilitation discharge: 93.3 ± 17.8 vs 98.8 ± 15.9, *P* < 0.001) in bivariable analysis. This association did not remain significant after adjustment for socio-demographics, cognitive and affective impairment, comorbidity, as well as functional status at admission.

## Discussion

This study highlights that unplanned transfers from rehabilitation to acute care occurred in about one in six stays, within the first week in almost half of the cases, and mostly because of infectious, cardiac, digestive, and trauma complications.

An original contribution of this study is to show that, when a transfer occurs, the reason for this transfer is more likely to lie within the same initial diagnostic category for orthopedic, trauma, and respiratory problems than in other initial diagnostic categories. This information could prove useful to better anticipate the potential type of complications according to the diagnostic category that motivates the initial admission to rehabilitation.

Results from the present study also extend previous observations about infectious and cardiac complications as the most frequent causes of transfers [1, 3] in showing different chronology over the rehabilitation stay. Indeed, cardiac problems accounted for almost a quarter of “early” unplanned transfers but only a sixth (15%) of “later” transfers. Inversely, infections ranked higher in “later” than in “early” unplanned transfers. Overall, this unique information about the precise timing of unplanned transfers and their specific causes certainly enhance our knowledge and could prove helpful to better anticipate unintended complications in older patients admitted to rehabilitation. In particular, the delayed timing of infectious complications further emphasizes the need for strong infection control measures in this setting.

Another significant contribution of this study is to extend information about patient’s characteristics associated with the risk of unplanned transfers. Older patients and those admitted for gait impairment were significantly less likely to experience an unplanned transfer, whereas men, patients with higher comorbidity, and functional impairment before their initial admission to acute care, as well as at admission to rehabilitation, were more likely to experience such transfer.

Higher odds of unplanned transfers among men have been inconsistently reported in similar previous studies, although these associations did not persist when potential confounders were taken into account [1, 2, 4, 34]. Several hypotheses can be proposed that relates to altered presentation in women with more frequent atypical symptoms and/or a lower pain threshold that all interfere with health professionals’ assessment [35, 36]. Alternatively, as men are less likely to be widowed, the presence of a partner may play a role, as suggested by a previous observation of an independent positive association between living with a partner and unplanned transfers [1]. In the present study this latter association (i.e. of living situation and transfer) did not however remain significant after adjustment. Overall, these heterogeneous findings may reflect more complex underlying relationships between healthcare utilization and socio-demographic or family-related factors that should be further explored in the future.

The inverse association between older age and unplanned transfers is more intriguing and has not been previously reported in similar studies [1, 2, 4]. The persistence of this association even after adjustment for comorbidity and functional status could raise some concern about potential ageism, with some transfer possibly denied to very old patients based solely on their age [37]. Unfortunately, we were unable to further investigate this hypothesis and determine from medical records these patients’ wishes about transfers. Indeed, it is possible that older multimorbid patients included in this study were more likely to express wishes against a potential transfer to acute care. It is also possible that some residual confounding remained.

This work also highlights that transferred patients were at higher risk of dying than other patients, even when controlling for several risk factors for death, such as age and the severity of comorbidities. Unplanned transfer should thus be considered as an indicator of a patient’s guarded prognosis. Nevertheless, the relatively high proportion of transferred patients who returned to rehabilitation still suggest that the decision was likely appropriate, provided it reflected patient’s wishes.

Finally, our results also contribute new information on the potential negative impact of unplanned transfers on patients’ functional recovery, as observed in some previous studies [2, 38, 39]. In the present study, transferred patients were indeed discharged with poorer functional performance at the end of their rehabilitation stay than patients without unplanned transfer. However, this association did not remain once controlling for functional status at admission, as well as for other predictors of functional recovery, such as age, cognitive and affective impairments. In other words, these patients’ worse health and functional status at baseline better predicted their poor functional recovery than the transfer. Indeed, despite the differences in functional performance between patients with and without unplanned transfer, the proportion discharged to their home was similar. In contrast, a previous study showed that patients with unplanned transfers were significantly less likely to be discharged home, but this proportion was based on the total sample, including patients who died and those who remained hospitalized in acute care without coming back to rehabilitation [2]. Overall, it remains difficult to disentangle the effect of the underlying disease and resulting health instability from the effect of the transfer itself.

### Strengths and limitations

A strength of the present study is its large sample size that resulted in a substantial number of unplanned transfers, allowing to investigate a wide range of patient’s characteristics associated with these transfers. An additional strength is the thorough identification of unplanned transfers and to determine their causes with extensive abstraction of discharge summaries from both rehabilitation and acute care. In particular, this detailed process allowed to exclude elective transfers, a limitation that is not always acknowledged in previous studies.

A limitation relates to the incidence rates and causes of acute transfers that were determined based on all stays during the two-year study period, with some patients represented more than once. As patients with multiple stays are likely to be in poorer health, they may artificially inflate transfer incidence. Of note, between March and June 2020, infections with Covid-19 (*n* = 13) may have increased the total number of infections (*n* = 59). However, this was likely counterbalanced by the quasi-absence of influenza cases. Another limitation is our inability to investigate whether some unplanned transfers were potentially avoidable. Finally, as the study was conducted in a single hospital, generalizability of the findings to other settings might be limited.

## Conclusion

Unplanned transfers occurred in about one in six older patients and were associated with poorer outcomes in terms of functional recovery and death. These patients were more likely to be men, with more severe comorbidity and functional impairment. Future studies should investigate whether interventions targeting these patients may prevent unplanned transfers and mitigate their death risk.

## Data Availability

Data are not publicly available. The datasets used during the current study are available from the corresponding author on reasonable request.

## Abbreviations

ADLs: Activities Of Daily Living (basic: BADLs, and instrumental: IADLs)
CIRS: Cumulative Illness Rating Scale
ED: Emergency Department
FIM: Functional Independence Measure
